# The Potential of Sheep or Camel Milk Constituents to Contribute to Novel Dressings for Diabetic Wounds

**DOI:** 10.3390/ijms242417551

**Published:** 2023-12-16

**Authors:** Zuzanna Flis, Piotr Szatkowski, Kinga Pielichowska, Edyta Molik

**Affiliations:** 1 Department of Animal Nutrition and Biotechnology, and Fisheries, Faculty of Animal Science, University of Agriculture in Krakow, Al. Mickiewicza 24/28, 31-059 Krakow, Poland; 2 Department of Biomaterials and Composites, Faculty of Materials Science and Ceramics, University of Science and Technology in Krakow, Al. Mickiewicza 30, 30-059 Krakow, Polandkingapie@agh.edu.pl (K.P.)

**Keywords:** sheep milk, camel milk, bioactive compounds, bioactive natural products, anti-inflammation, diabetic wounds, infectious diseases

## Abstract

Impaired wound healing is a complication of diabetes, which constitutes a serious problem in clinical practice. Currently, there is a high demand on the market for local treatment options for difficult-to-heal wounds caused by diabetes. The development of dressings that accelerate wound healing has recently been the subject of much research. Sheep and camel milk is gaining importance due to the content of many bioactive substances with health-promoting effects, such as insulin, LF, proline, or CLA. Sheep and camel milk proteins are a promising source of insulin, antidiabetic, and antihypertensive peptides. Numerous studies show that local administration of insulin has a significant impact on the healing of diabetic wounds. Sheep and camel milk, due to the highest LF content among ruminants, reduces autoimmune inflammatory processes and protects against bacterial and viral infections in the wound environment. Sheep’s milk has the highest content of proline and CLA, and their addition to a hydrogel dressing can help in the development of an effective dressing material. The production of hydrogel dressings containing sheep and camel milk, which are naturally rich in the bioactive substances presented in this review, may be a promising step in the market of specialized dressings for difficult-to-heal diabetic wounds.

## 1. Introduction

The special biological properties of sheep and camel milk result from the high content of biologically active substances that are beneficial to health. Milk bioactive substances are also very popular due to their natural origin and lack of toxicity. These substances are used in the treatment of skin diseases, restoring immunity, and fighting cancer. Many years earlier, sheep’s milk was used in traditional medicine to treat difficult-to-heal wounds, animal bites, and purulent skin lesions [1]. Currently, dairy products are increasingly used in the treatment of dermatological diseases to support the healing of chronic wounds, accelerate tissue regeneration, and alleviate inflammatory lesions due to their moisturizing, protective, smoothing, soothing, and antiaging effects [2]. Inadequate diet, repeated injuries, and diabetes are just some of the factors that seriously impair the wound healing process. Lifestyle diseases, which include, among others, cardiovascular diseases, obesity, diabetes, and cancer, cause the death of approximately 40 million people each year, which constitutes approximately 70% of all deaths in the world. Currently, over 2 million people in Poland suffer from diabetes, of which about 25% do not know about it. It is expected that in the next 15–20 years the number of people with diabetes in our country will double. It is worth emphasizing that the risk of developing an ulcer over the lifetime of a diabetic patient is 12–25%, and the probability of amputation in diabetic patients is 30–40 times higher than in people without diabetes. Moreover, wound healing is impaired in diabetic patients. Therefore, the search for medicinal products of natural origin that would contribute to improving the patient’s health is important not only from a socio-economic point of view but also from a clinical perspective.

## 2. Methodology

At the initial stage of preparing this review, the topic was selected. After searching the available literature, it was concluded that there is a need to gather in a review article up-to-date information on the possibilities of using sheep and camel milk in dressings for difficult-to-heal diabetic wounds. Then, by selecting the appropriate keywords, the following scientific databases were searched: PubMed, Web of Science, Science Direct, Scopus, Google Scholar, and Google. The last accession to the online databases was conducted in October 2023. When selecting scientific articles, the aim was to ensure that over 50% of the publications were published within the last 5 years. The search results were then reviewed and the information analyzed, categorized, and presented in sections to effectively address the scope of this review. 

## 3. Diabetic Wounds as a Growing Society Problem 

Diabetes is a chronic metabolic disease that affects the body’s glucose metabolism mechanism [3]. It is characterized by glycemic dysregulation due to the body’s inability to secrete insulin (type 1 diabetes) or insulin resistance (type 2 diabetes) [4,5]. According to the International Diabetes Federation, worldwide in 2021, 537 million adults (20–79 years old) were living with diabetes [6]. This number is projected to increase to 643 million by 2030 and 783 million by 2045 [6]. Type 2 diabetes is the most common type of diabetes, accounting for about 90% of all cases of this disease [6]. Type 2 diabetes is a complex disease and leads to many complications, such as kidney failure, heart disease, blindness, and ulcers, followed by amputation of lower limbs. Impaired wound healing is a complication of diabetes and a serious problem in clinical practice [7]. As many as 15% of people with diabetes will develop foot ulcers and wounds, and 3% will require lower limb amputation [8]. Diabetic feet are wounds infected mostly with polybacterial microorganisms characterized by high resistance to antimicrobial agents [9]. Injury triggers a cascade of events aimed at repairing damaged tissue and restoring skin integrity [10]. Depending on the size of the wound and the degree of damage, extensive physiological and metabolic changes may occur, causing complications in the healing process and a decrease in patients’ immunity [10]. Infected wounds in diabetic patients are associated with high costs, morbidity, and mortality [9]. This disease and its complications, including diabetic wounds and ulcers, are considered a global epidemic of the 21st century. Based on these facts, there is a high demand for supportive topical treatment options [9]. Natural drug alternatives, including food-derived peptides, are currently being explored to prevent and aid in the treatment of complications [3]. It is also worth mentioning synthetically produced compounds, such as iminosugars and sugar derivatives, which are a group of compounds imitating carbohydrates. They have anticancer, antidiabetic, antiviral, and antibacterial properties. The synthesis of ring sugars, which may be potential glycosidase inhibitors, is currently an important area of research in the discovery of modern antidiabetic drugs [11,12]. However, much attention is focused on natural alternatives such as bioactive peptides to support the treatment of type 2 diabetes and associated difficult-to-heal wounds [3]. In this aspect, sheep and camel milk can be perfect, as they have many health-promoting properties for the body due to their rich profile of bioactive substances and can be used to produce hydrogel dressings.

## 4. Characteristics of Hydrogel Dressings

Hydrogels are characterized by good ductility, high water content, and favorable oxygen transport, which makes them one of the most promising materials for wound dressings [13]. Additionally, nanomaterials exhibit improved biodegradability, biocompatibility, and colloidal stability in wound healing and may play a role in promoting healing due to their properties or as carriers of other bioactive substances [13]. As Zhang et al. (2023) point out, hydrogels are safe for health and, what is more, they can also have a positive effect on the healing process of all types of wounds [13]. It is a matter of selecting an appropriate, safe material that will coat the hydrogel and have health-promoting potential. Taking into account the content of bioactive substances in camel and sheep milk, it is worth thinking about the innovative use of these substances in medical materials. The possibility of using bioactive substances of these products in hydrogel dressings may have a positive impact on wound recovery. It should be noted that sheep and camel milk is a food product and, therefore, does not have any toxic effects on the body. Additionally, sheep and camel milk is characterized by high stability; the method of milk processing needed to create dressings (e.g., freeze-drying) is safe and does not reduce the nutritional value of the milk [14]. According to research, the milk processing process may even increase its bioactive properties. According to researchers, milk processing, especially the fermentation process, increases the antithrombotic properties of sheep’s milk polar lipids against platelet-activating factor (PAF) [15] and thrombin-induced platelet aggregation [16]. Serafeimidou et al. (2013) determined changes in conjugated linolenic acid (CLA), concentration, fatty acid profile, and chemical composition of cow’s and sheep’s milk yogurts produced using traditional methods during 14-day storage at 5 °C [17]. During the storage period, no significant changes were observed in the content of SFA, MUFA, and PUFA in sheep’s milk yogurts. Moreover, the CLA content decreased significantly at the end of storage in cow’s milk yogurts, while it increased significantly in sheep’s milk yogurts [17]. However, as reported by Wendorff, (2001), in order to maintain high quality of milk, it is recommended to freeze the milk immediately after collection and store it at a temperature of −20 °C or lower for no longer than 6–12 months [18]. The introduction of hydrogel dressings with camel and sheep milk allows for the innovative use of bioactive substances in medical materials. It allows for local, targeted delivery of insulin directly to the wound, which improves the healing process. Hydrogel dressings with the addition of milk bioactive substances can be used for various types of wounds and individual patient needs, which increases their effectiveness. For economic reasons, if a targeted dressing accelerates the healing process and reduces the risk of complications, it contributes to reducing healthcare costs. Research on the development of hydrogel dressings with camel and sheep milk has great market potential because it offers a product based on natural bioactive substances. Ensuring proper moisture at the bottom of the wound and supporting its self-renewal processes are tasks in which biologically active dressings play a key role. Traditional methods of dressing wounds with dry gauze dressing are being abandoned. A dry compress does not serve any additional function beyond the external wound cover. Moreover, when changing a dry dressing, trauma to the built-up tissue structures may occur, which significantly prolongs the overall healing time. Currently, alginate, foam, and hydrogel dressings containing silver, manuka honey, paraffin, or activated carbon are very popular. In recent years, consumers have become more interested in natural products that have health-promoting properties. Cosmetic products containing sheep or goat milk currently on the market are also gaining great popularity and consumer trust. Therefore, the dressings containing sheep and camel milk proposed in this article, due to the fact that they are natural and safe and show biological activity, may prove to be a breakthrough in the market of dressings for difficult-to-heal wounds. The hydrogel dressings market is large and constantly developing, and the proposed bioactive substances from sheep and camel milk have confirmed medicinal properties (e.g., insulin). The hydrogel dressings market is worth approximately USD 2.5 billion and is growing at a rate of approximately 8% annually, driven by increasing consumer awareness of the health benefits of hydrogel dressings and interest in natural substances in medical products. The growing demand for hydrogel dressings for difficult-to-heal wounds results from demographic conditions (aging population), an increase in the number of people with chronic diseases, such as diabetes and comorbidities, e.g., extensive wounds.

## 5. The Effect of Insulin

Both sheep and camel milk, compared with cow’s milk, are rich in insulin, which is particularly important in the wound healing process [19]. Insulin is a peptide hormone and growth factor that regulates glucose levels in the blood. Recently, there has been an increased interest in research examining the impact of camel milk consumption on the glycemic status of animal models and diabetic patients. Camel milk reduced the level of glucose and glycosylated hemoglobin in the blood [20]. It was found that camel milk does not form a coagulum in the stomach and milk insulin can be absorbed through the entire digestive tract unchanged and then absorbed in the intestine [20]. Additionally, other camel milk proteins can also interact with the insulin receptor and contribute to the regeneration of pancreatic cells through antioxidant and anti-inflammatory effects [20]. Insulin may support the healing of damaged skin by regulating oxidative and inflammatory reactions and stimulating cell migration. Numerous studies show that local administration of insulin has a significant effect on the healing of diabetic and non-diabetic wounds, burn wounds, and acute and chronic wounds [21,22,23]. Topical application of insulin can reverse the impaired inflammatory response in the diabetic wound environment and accelerate the wound healing process in diabetics [24]. Studies in rats with diabetic wounds have shown that insulin-modulated macrophages switch from a pro-inflammatory (M1) to an anti-inflammatory (M2) macrophage phenotype [24,25]. Both in vivo and in vitro studies confirmed that insulin restores the phagocytosis function and promotes the phagocytosis-induced apoptosis process in neutrophils [24]. The anti-inflammatory effects of insulin have been shown to be mediated by the PI3K/Akt/Rac-1 and PPAR-γ signaling pathways [25]. Additionally, insulin inhibits high glucose-induced transcriptional activation of p38, NF-κB, and STAT1 by activating Akt-Rac-1 signaling [25]. Moreover, insulin has anti-inflammatory effects by increasing PPAR-γ expression and inducing P38 (Ser112)-mediated PPAR-γ dephosphorylation [25]. In diabetic patients, local injection of insulin at the wound site promotes wound healing also by improving the growth of granulation tissue [22,23]. However, injection into sensitive tissue is painful for patients and difficult to perform on their own, while in the case of medical creams with insulin the effectiveness of absorption may be problematic due to the abrasion of the preparation from the wound surface. Therefore, due to its ease of application and use, the integration of insulin into dressings may be groundbreaking. Research by Zhao et al. (2017) revealed that the incorporation of insulin and fibroblasts into a hydrogel dressing can promote neovascularization and collagen deposition and improve the healing process of diabetic wounds [26]. The use of biomaterials as wound dressings has attracted more and more attention in recent years, and hydrogels filled with bioactive substances have the potential in wound treatment as a targeted therapeutic system [26]. Li et al. (2021) produced a bioactive hydrogel dressing enriched with insulin and applied it to diabetic rats at the wound site [27]. Studies have shown that the dressing shortened the inflammatory phase, enhanced the formation of granulation tissue, promoted collagen deposition, accelerated epithelial regeneration and neovascularization, and delayed or even improved peripheral neuropathy, thus significantly accelerating the healing of diabetic foot ulcers [27]. Camel milk can be used in the treatment of type 1 and type 2 diabetes due to the high level of insulin—approximately 52–59 units/liter and insulin-like substances (Table 1) [28]. Sheep milk proteins are also a promising source of insulin, antidiabetic, and antihypertensive peptides [19,29]. For this reason, the design and production of hydrogel dressings containing sheep and camel milk, which are naturally rich in insulin, may be a promising step on the market of specialized dressings for difficult-to-heal wounds. 

## 6. The Importance of Camel Milk Whey Proteins

Recently, natural antioxidants have gained great popularity in medicine and pharmacy due to the fact that they are safe, effective and inexpensive [34]. Camel whey proteins are considered a strong and natural antioxidant because they reduce oxidative stress, improve the functioning of the immune system, and increase the level of glutathione [34]. The ability of sheep whey protein to scavenge free radicals in vitro was also determined [35]. Research by Kerasioti et al. (2014) showed that the protein effectively removed 2,2-diphenyl-1-picrylhydrazylic acid (DPPH), 2,2′-azino-bis(3-ethylbenzothiazoline-6-sulfonic acid (ABTS), and hydroxyl radicals, increasing glutathione levels [35]. Research by Ebaid et al. (2011) showed that dietary supplementation of whey protein obtained from camel milk accelerates the wound healing process in diabetic mice [36]. Whey proteins include many components such as lactoferrin, lactalbu-min, lactoglobulins, lactoperoxidase and lysozyme, and immunoglobulins [34,37]. These ingredients may support diabetic wound healing by reducing blood glucose levels, oxidative stress, growth factor levels, and inflammation [34,37]. This was also confirmed by subsequent research by Ebaid et al. (2015), which showed that peptides extracted from camel milk can be considered effective antioxidants and immune system stimulators that induce oxidative stability and accelerate wound healing in diabetic model rats [38]. These properties make camel milk a potential agent supporting wound healing in people with diabetes [38]. Camel milk whey proteins regulate TNF-α and cell death receptor (Fas) mRNA expression and then support the closure and healing of diabetic wounds [39]. Moreover, supplementation of camel milk whey proteins in diabetic mice improves and accelerates the healing of skin wounds by reducing oxidative stress and restoring the levels of pro-inflammatory cytokines and β-defensin [40].

## 7. The Effect of Lactorefin

LF is a glycoprotein that supports many biological processes related to wound healing and tissue regeneration. It has anti-inflammatory effects, directly promotes the formation of granulation tissue, stimulates the proliferation and migration of fibroblasts and keratinocytes, and enhances the synthesis of collagen and hyaluronan [41]. One of the most important factors hindering the healing process of skin wounds in diabetics is the presence of numerous bacteria in the damaged tissue [42]. LF has a high affinity for iron ions, this feature makes it bacteriostatic because it binds the element needed for the development of bacteria, especially gram-negative ones, such as *Escherichia coli*, *Helicobacter pylori*, *Salmonella enterica sv Typhimurium*, *Aggregatibacter actinomycetemcomitans*, and other types of *Bacillus* spp., *Staphylococcus aureus*, *Listeria monocytogenes*, *Mycobacterium tuberculosis,* and *Streptococcus mutans*. The LF content in sheep milk is 0.7–0.9 g/kg, in camel milk 0.2–0.9 g/kg, and in cow milk 0.02–0.5 g/kg [31] (Table 1). Sheep milk, due to the highest LF content [43], reduces autoimmune inflammatory processes and protects against bacterial and viral infections [44]. Camel milk is rich in active peptides, LF, zinc, and mono- and polyunsaturated fatty acids [45]. For this reason, camel milk has antibacterial but also antioxidant and antidiabetic properties [45]. The development of innovative wound dressings with highly effective antibacterial properties and accelerating wound healing has recently been the subject of many studies. Chouikhi et al. (2021) isolated a probiotic strain, *Lactiplantibacillus* plantarum LC38, from camel milk [46]. Studies have shown that the application of Lactiplantibacillus plantarum LC38 to the wound site of a diabetic rat significantly enhanced the healing process and accelerated wound closure 14 days after wound induction [46]. As the authors point out, the strong wound healing potential may be due to the antimicrobial effect of camel milk [46]. Hewitt et al. (2019) created bioactive scaffolds supporting skin regeneration containing bioactive milk proteins such as LF [47]. Biological activity determined in vitro using human keratinocytes (HaCaT) and normal human dermal fibroblasts showed significantly increased cell growth [47]. Studies have shown that the addition of milk bioactive substances increases the biological activity of the resulting scaffolds and they can be used for deep regeneration of skin tissues [47]. Wang et al. (2022) synthesized a hydrogel containing LF and lithium magnesium silicate, which effectively promotes wound healing [48]. The production of a hydrogel dressing with the addition of milk, especially sheep milk, which is naturally the richest in LF, is an extremely promising prospect. This is a huge opportunity to improve the comfort of treatment of patients with burn or diabetic wounds.

## 8. The Importance of Proline

Bioactive peptides derived from milk proteins, during fermentation processes and hydrolysis reactions by proteolytic enzymes, play a protective role for body cells by releasing amino acids such as proline [49]. Angiotensin I-converting enzyme (ACE) inhibitors are widely used to treat hypertension. Food-derived, ACE-inhibiting peptides may be a good alternative to synthetic drugs. In the study by Mudgil et al., (2023), it was found that a valuable source of peptides with high antihypertensive potential are goat and camel milk peptides released during the fermentation process [50]. Moreover, Alhaj, 2016 reported that all ACE-inhibiting camel milk peptides identified in the study contained at least one proline residue at the C-terminal position [51]. This presence of proline results in greater effectiveness in the ACE inhibitory effect [51]. The main extracellular protein in the granulation tissue of a healing wound is collagen [42]. Its synthesis increases rapidly and immediately after injury at the wound site, thus ensuring the strength and integrity of the tissue matrix [42]. Proline and hydroxyproline are the main amino acids present in collagen [52]. Proline is essential for active collagen synthesis, and its availability at the wound site can accelerate collagen synthesis [53]. Physiologically, during the first 10 days of healing, proline levels in the wound are 30–50% higher than in plasma, suggesting that proline import into the wound occurs actively or that proline biosynthesis occurs in the wound environment [54]. Proline also protects tissue against damage caused by free radicals and reactive oxygen species thanks to its antioxidant potential [52]. Local administration of proline has a beneficial effect on the healing process of open postoperative wounds in rats [52]. It was observed that wounds treated topically with proline healed very quickly, as evidenced by an increased rate of epithelial regeneration and wound contraction [52]. Milk proteins are characterized by a balanced amino acid composition, which promises innovative functional products. The addition of proline to the hydrogel may help in the development of an effective dressing material used in patients with diabetic wounds. Research showed that hydrogels with the addition of proline showed better antioxidant activity, increased cytological compatibility, and cell proliferation [53]. Moreover, hydrogel dressings with the addition of proline are more thermally stable than other hydrogels, which is important from a technological point of view [53]. Both sheep’s milk and camel’s milk are characterized by a high content of proline, which also affects the synthesis of hemoglobin. Also, yogurts made from sheep and camel milk had the highest proline content, compared to the milk of other ruminants [55]. Research on the analysis of the chemical composition and nitrogen of milk fractions of various ruminant species showed that the highest proline content was in sheep milk (537 mg/100 g of milk) compared to the milk of other ruminants (cow milk 302.23 mg/100 g of milk, buffalo 370 mg/100 g of milk, and goat 273 mg/100 g of milk) [32] (Table 1). Studies show that camel milk hydroxyproline promotes regenerative tissue repair in streptozotocin-induced diabetic mice [42]. Higher antioxidant activity was observed in camel milk than in cow milk, probably due to structural differences and the presence of higher proline content in camel milk caseins [49,56,57]. This can be explained by the presence of a higher proline content in the primary structure of camel milk caseins than in cow milk [49,56,57]. This was also confirmed by research by Rafiq, (2016), which showed that camel whey proteins had the highest proline content (129 mg/g) (Table 1) [33].

## 9. The Importance of Milk Lipids

The milk fat globule is a very important structural element of milk with technological and biological significance. The camel milk fat globule membrane has antiadhesive and antibacterial properties [58]. As reported by Mansour et al. (2015), a cream containing 25% sheep milk fat globules accelerates the healing of burns and reduces the severity of inflammation, accelerates wound contraction, minimizes scars, and improves the treatment of burn wounds [59]. Other lipids with an important biological role are butyric acid, CLA, sphingolipids (components of fat molecular membranes), and carriers of vitamins A, D, E, K, and carotenoids. The content of short-chain fatty acids, such as butyric acid, caproic acid, caprylic acid, and capric acid, is 2–3 times higher in sheep’s milk than in goat’s milk. Omega-3 acids include eicosapentaenoic acid (EPA) and docosahexaenoic acid (DHA). Additionally, polyunsaturated acids protect and strengthen the endothelium of blood vessels, which is important in the wound healing process. Polyunsaturated fatty acids also reduce the expression of genes responsible for inflammatory reactions and suppress the secretion of cytokines, eicosanoids, and reactive oxygen species. Among ruminants, sheep’s milk is the richest in CLA, which has many health-promoting properties [60] (Table 1). The most biologically active isomer of CLA is cis-9, trans-11, which inhibits the occurrence and development of skin cancer [61]. CLA supplementation accelerates skin wound healing by regulating antioxidant and anti-inflammatory functions [62]. Research by Tang et al. (2020) in a mouse model of atopic dermatitis showed that topically applied CLA has a protective effect on the skin due to the regulation of skin barrier function and the inflammatory response [63]. Topical application of CLA significantly inhibited pro-inflammatory cytokines, including interleukin (IL)-6, IL-1β, tumor necrosis factor (TNF)-α, and the expression of the pro-inflammatory enzymes inducible nitric oxide synthase (iNOS) and cyclooxygenase-2 (COX-2) in mouse dermatitis [63]. The anti-inflammatory effects of CLA have been associated with the inhibition of DNFB-stimulated p65 phosphorylation of IκBα and NF-κB in mouse skin [63]. Moreover, topical application of CLA resulted in repair of the skin barrier, including maintaining a balanced skin pH and increasing its hydration [63]. The highest CLA content in sheep’s milk makes it possible to investigate the surface effect of sheep milk’s CLA on the wound healing process, including diabetic wounds. The presented research may be particularly important when designing dressings coated with bioactive substances, in particular sheep’s milk, for difficult-to-heal wounds caused by diabetes. 

## 10. The Importance of Sheep’s Milk Polar Lipids

Sheep’s milk is an important source of polar lipids (PLs), which in particular are the main components of biological membranes. PLs perform essential functions in energy storage, both as structural components of cell membranes and signaling molecules [61,64]. PLs play a major biological role in the structure of the epidermis and hair. They ensure intercellular cohesion and protect the skin against the penetration of harmful compounds. They also play a structural role and ensure the mechanical properties of the epidermis [65,66]. PLs in milk are mainly located in the membrane of milk fat globules (MFGs). Currently, PLs from animal sources are gaining importance because they are rich in highly bioactive compounds that have a significant impact on cell metabolism and regulation [67]. Phospholipids and sphingolipids are the two main classes of PLs in milk [68,69]. Phospholipids and sphingolipids in milk are becoming more and more popular due to their biological and technological values. Studies by Lagutin et al. (2022) showed that sheep’s milk is the richest source of phospholipids [70]. Studies by Fluhr et al. (2001) have shown that free fatty acids produced from phospholipids regulate the integrity and cohesion of the stratum corneum [71]. Sphingolipids and their derivatives are bioactive compounds with bacteriostatic properties and are associated with modulating inflammatory responses [67]. PL sheep’s milk has many health-promoting properties, which are becoming increasingly important in the nutraceutical and pharmaceutical industries. Current market and research trends concern the use of bioactive PLs in sheep’s milk and the development of products targeted at difficult-to-heal wounds, which may be particularly important in patients with diabetic feet.

## 11. The Importance of Antioxidant Substances

Milk contains many antioxidants, mainly vitamins A, D, E, and C, coenzyme Q10, calcium, magnesium, iron, zinc, CLA, and LF. Vitamin A is essential for the development of epithelial tissue and proper wound healing [54]. Vitamin A stimulates the process of angiogenesis and collagen synthesis as well as the growth of epithelium, fibroblasts, and granulation tissue [72]. Camel milk contains the most vitamin A (0.36 mg/100 g), compared to sheep milk (63 μg/100 g of milk) (Table 2) and cow milk (39 μg/100 g of milk) [31,73]. The vitamin D content in sheep and camel milk is 0.18 μg/100 g of milk and 0.9 μg/100 g of milk, respectively (Table 2) [31]. Vitamin E modulates the expression of connective tissue growth factor (CTGF)53 and regulates gene expression and transcription, thereby helping to protect wounds against infection [74]. The content of vitamin E in sheep and camel milk is 120 μg/100 g of milk and 89 μg/100 g of milk, respectively (Table 2) [31]. Vitamin C also plays an important role as it reduces wound inflammation and is necessary for fibroblast maturation and angiogenesis. Additionally, it is crucial in the process of creating cross-links between collagen fibers, which promotes collagen synthesis [75]. Vitamin C also enhances ceramide synthesis, creating strong barrier lipids in the epidermis. The vitamin C content in camel and sheep milk is the highest among all ruminants and amounts to 5.38 mg/100 g of milk and 4.49 mg/100 g of milk, respectively (Table 2) [31,73]. Coenzyme Q10 is also an important antioxidant, which, through interactions with membrane proteins, stabilizes cell membranes, increasing their resistance and reducing the amount of substances such as water or ions leaving the cell. Extracellular calcium affects the skin wound healing process and can be used as a new biomolecule modulating this process. Sheep milk is characterized by the highest content of calcium (196 mg/100 mL of milk) and magnesium (19 mg/100 mL of milk) (Table 2) among the milk of other ruminants [31]. Navarro-Requena et al. (2018) conducted a study to investigate the effect of extracellular calcium on skin fibroblasts cultured in vitro [76]. Extracellular calcium supplementation has been found to increase cell metabolic activity, migration, MMP production, collagen synthesis, and cytokine release, and also reduces the ability of cells to contract [76]. Yin et al. (2021) developed a multifunctional patch based on organic magnesium, which significantly accelerates the healing of diabetic wounds [77]. Due to the highest iron (0.45 mg/100 g) and zinc (1.68 mg/100 g) content (Table 2) [31,73], camel milk is extremely important in wound healing. Studies show that zinc supplementation in patients with diabetic foot ulcers has a beneficial effect on ulcer size parameters and metabolic profile [78]. Moreover, zinc oxide nanoparticles also have a strong antibacterial effect and have a significant impact on the healing process of diabetic wounds due to their antioxidant and anti-inflammatory effects [79]. People with iron deficiency experience difficulties already at the initial stage of the healing process. Das et al. (2019) produced biocompatible and biodegradable dressing materials containing a Fe–Cu nanocomposite with broad-spectrum antibacterial properties [80]. Moreover, it has also been found that the resulting dressings help in the healing of infected diabetic wounds and may be used in the treatment of other infectious wounds [80]. The presented research presents a promising perspective for the use of bioactive antioxidant substances from sheep and camel milk to develop dressings dedicated to patients with diabetic wounds.

## 12. Conclusions

Nowadays, consumers are interested in natural products with health-promoting properties. Due to the content of many biologically active substances, sheep and camel milk is gaining importance. Therefore, an extremely promising and innovative solution is the use of the milk of these animals to produce specialized dressings for difficult-to-heal and chronic wounds. The use of biomaterials with sheep and camel milk is an innovative use of bioactive substances, which may allow for targeted delivery of insulin directly to the wound, which may facilitate the healing process. Additionally, this type of dressings can be adapted to different types of wounds and individual patient needs, which increases their effectiveness. For economic reasons, a targeted innovative dressing, if it accelerates the healing process and reduces the risk of complications, also contributes to reducing healthcare costs and patients’ health comfort. The evidence presented in this review shows that sheep and camel milk, due to the high content of bioactive substances such as, among others, LF, proline, or CLA can be used as an alternative element of dressing material to accelerate tissue repair in diabetic wounds.

## Figures and Tables

**Table 1:** Contents of selected bioactive substances in sheep and camel milk.

Compounds	Sheep Milk	Camel Milk
Insulin	0,44 µg/L of milk [30]	52–59 units/L of milk [28]
LF (g/kg of milk)	0.7–0.9 [31]	0.2–0.9 [31]
Proline	537 mg/100 g of milk [32]	129 mg/g of casein [33]
CLA (% total milk fatty acid)	0.8 [31]	0.4 [31]

**Table 2:** Contents of selected antioxidant substances in sheep and camel milk.

Compounds	Sheep Milk	Camel Milk
Vitamin A	63 (μg/100 g) [31]	0.36 (mg/100 g) [73]
Vitamin D (μg/100 g)	0.18 [31]	0.9 [31]
Vitamin E (μg/100 g)	120 [31]	89 [31]
Vitamin C (mg/100 g)	4.49 [31]	5.38 [73]
Calcium	196 (mg/100 mL) [31]	112.93 mg/100 g [73]
Magnesium	19 (mg/100 mL) [31]	9.65 mg/100 g [73]
Iron	0.06 (mg/100 mL) [31]	0.45 mg/100 g [73]
Zinc	0.55 (mg/100 mL) [31]	1.68 mg/100 g [73]

## Data Availability

Data are contained within the article.
